# Emotion Discrimination Using Spatially Compact Regions of Interest Extracted from Imaging EEG Activity

**DOI:** 10.3389/fncom.2016.00055

**Published:** 2016-07-20

**Authors:** Jorge I. Padilla-Buritica, Juan D. Martinez-Vargas, German Castellanos-Dominguez

**Affiliations:** ^1^Signal Processing and Recognition Group, Department of Electrical and Electronic Engineering, Universidad Nacional de ColombiaManizales, Colombia; ^2^Diseño Electrónico y Técnicas de Tratamiento de Señal, Universidad Politecnica de CartagenaCartagena, Spain

**Keywords:** emotion classification, electroencephalograph, brain mapping, LORETA, MSP, region of interest

## Abstract

Lately, research on computational models of emotion had been getting much attention due to their potential for understanding the mechanisms of emotions and their promising broad range of applications that potentially bridge the gap between human and machine interactions. We propose a new method for emotion classification that relies on features extracted from those active brain areas that are most likely related to emotions. To this end, we carry out the selection of spatially compact regions of interest that are computed using the brain neural activity reconstructed from Electroencephalography data. Throughout this study, we consider three representative feature extraction methods widely applied to emotion detection tasks, including Power spectral density, Wavelet, and Hjorth parameters. Further feature selection is carried out using principal component analysis. For validation purpose, these features are used to feed a support vector machine classifier that is trained under the leave-one-out cross-validation strategy. Obtained results on real affective data show that incorporation of the proposed training method in combination with the enhanced spatial resolution provided by the source estimation allows improving the performed accuracy of discrimination in most of the considered emotions, namely: dominance, valence, and liking.

## 1. Introduction

The design of reliable systems and devices, which can recognize, interpret, and process human emotions, still poses a challenging task. As long as the latent factors that generate emotions are unobservable, the main problem is how to rely on visible manifestations of emotions to reproduce and verify them. There are several objective measures, proposed for estimating unseen psychological activity, that are often related to expressive movements (facial expressions, gestures, movements of limbs, vocal emission, etc.), where facial expression analysis is one of the most frequently addressed themes (Marrero-Fernandez et al., 2014). Although these models can be more manageable to implement, human beings considerably vary in the manner which they manifest motivational states through movement patterns (Wentzel and Wigfield, 2009). Moreover, humans convey emotional information both intentionally and non-intentionally, producing consciously regulating or naturally suppressing emotional expressions. Therefore, derived motivational movement patterns may be too short for the goal of emotion recognition (Virvou et al., 2015).

As an option to expressive movements is the use of physiological components generated by the autonomic nervous system, such as electrocardiogram (Agrafioti et al., 2012), skin conductance (Muthu Meena et al., 2015), respiration and blood pressure (Dan-Glauser and Gross, 2015) to improve the accuracy and robustness of emotion recognition systems. In comparison with methods based on expressive movements, the responses of peripheral physiological signals tend to provide more detailed and complex information as an indicator for estimating emotional states (Wang et al., 2014). However, neuroscience has provided strong evidence that several cortical and subcortical regions (e.g., insula, prefrontal regions, thalamus, amygdala, hippocampus, basal ganglia) are implicated in emotional perception and regulation (Kober et al., 2008). Consequently, a growing number of studies have been enabled recently to engage and thus measure emotional functions using neuroimaging paradigms mostly through brain signals captured from the central nervous system such as electroencephalogram (EEG) and functional magnetic resonance imaging (fMRI). Nonetheless, the first technique is more desired in emotion recognition systems due to its non-invasiveness, cost effectiveness, and simple acquisition (Robinson and Robinson, 2009). Another aspect of consideration is the brain activity nature of emotional functions, which are partially localized in both space (some cortical and subcortical regions) and frequency (mostly upon the neural oscillation bands). Although EEG suffers from its poor spatial resolution and raised susceptibility to noise, this biosignal is a plausible approach in emotion recognition due to its provided high temporal resolution to investigate the brain dynamics (Liu et al., 2014).

Most of the research studies devoted to feature extraction put emphasis on the spectral power of theta, alpha, beta, and gamma bands (Jirayucharoensak et al., 2014). Due to the nonstationary behavior of EEG signals, the features, which aim at discrimination of time-series, are also extracted, including time-varying (Alvarez-Meza et al., 2015), time-frequency (Sirca et al., 2015), and nonlinear analysis methods (Liu and Sourina, 2013; Ahammed, 2015). Nevertheless, some issues remain open in characterizing EEG data for emotional recognition. Moreover, interpretation of extracted features from EEG sensor level is not straightforward due to a couple of basic reasons: the low spatial resolution provided by EEG recordings and the influence of field spread that severely corrupt data, i.e., neural activity travels from cortical to scalp surface through several head tissues (Schoffelen and Gross, 2009). To overcome this concern, some methods are focused on characterizing the connectivity networks of brain sources reconstructed from scalp EEG signals (Brookes et al., 2014), where usually, a realistic model of the brain cortex serves as the source space. However, some methodological aspects remain still open. Particularly, mapping solutions with a simplistic spatial structure are assumed to be favored regarding neurophysiological plausibility. Nonetheless, the simplest approaches are the minimum norm estimate that minimizes the overall power of the sources and Low-Resolution Tomography (LORETA) that explicitly enforces spatial smoothness of the sources, making either approach to producing poor spatial accuracy. Furthermore, it has also been argued that only a small fraction of the brain should be consistently activated in event-related experimental designs (Castaño-Candamil et al., 2015).

Consequently, the used mapping algorithm must provide an accurate location of those brain areas that are most related to the studied task (or *Regions-of-interest* – ROIs). Therefore, the ROIs identification can be performed relying on some prior knowledge about their association with the underlying research task. In other words, the evidence provided previously by other functional imaging studies is accepted as discussed in Schoffelen and Gross (2009). Additionally, some ROI selection methods have been proposed mainly based on anatomical constraints (Nordhjem et al., 2016), fostering brain areas with similar temporal responses (Edelman et al., 2016), or as part of a feature extraction procedure under a supervised classification framework, i.e., regarding the classification accuracy as proposed in Illán et al. (2011). Nevertheless, the widely allowed strategy is the determination of those reconstructed brain areas with a potent activity during the experimental task (Robinson and Robinson, 2009), even though determination of the best strategy of ROI selection for each task under consideration remains an open issue. Finally, once the ROIs are suitably selected, the feature extraction procedure is carried out over their time series, improving the low spatial resolution of raw EEG data, and avoiding the field spread effect (Toppi et al., 2016).

Although many features may be extracted from both the raw EEG data or the ROI time courses, their main restriction is how to extract discriminative features as much as possible since several features may not contain relevant information introducing redundancy (Verleysen and François, 2005). Consequently, extracted feature set may decrease classification accuracy. Therefore, an emerging challenge for emotion discrimination is how to tackle the problem of large training feature spaces. To this end, relevance analysis algorithms are used, by instance, the one developed in Alvarez-Meza et al. (2015) that is built on the eigenvalues and eigenvectors of the feature covariance matrix. In this case, the relevance measure is assumed to point out to the best input attributes that exhibit higher overall correlations to the estimated principal components. For validation experiments of effective emotion discrimination, SVMs are a powerful machine learning approach that has been successfully applied to solving problems with complex multi-class features, overcoming some of the drawbacks relating to over-fitting (López et al., 2011).

Under the aforementioned considerations, we present a new method for discrimination of emotions that is founded upon the extraction of relevant ROI time series from EEG data, yielding an improved accurate location of the brain areas related to emotion states. In order to map accurately the scalp recordings to the source space, we solve the ill-posed EEG inverse problem by employing the Multiple Sparse Priors Algorithm (MSP) as to encourage sparse and focal solutions (Friston et al., 2008). Further, the data-driven selection of the regions of interest is accomplished along with their respective time courses estimation, holding two main aspects: (i) An adequate selection of the brain areas should allow characterizing the brain activity directly related to the specifically studied task, and (ii) Quality of the estimated time courses biases the goodness of estimated features. Obtained results on real affective data show that incorporation of the proposed training method in combination with enhanced spatial resolution allows improving the performed accuracy of discrimination in most of the considered emotions, namely: Arousal, dominance, valence, and liking.

The rest of the paper is organized as follows: Section 2 describes materials and methods, the experimental setup is explained in detail in Section 3, this is followed by the discussion and concluding remarks in Section 4.

## 2. Materials and methods

### 2.1. Brain source activity with spatially localized solutions

In order to assess the needed connectivity analysis between distant brain regions, all measured EEG signals must be evaluated upon the source space, i.e., the scalp neural field recordings should be projected into brain volume that we carry out within the inverse problem framework. To this end, the measured EEG data are expressed through a multivariate linear model that incorporates a distributed source representation with fixed positions and orientations as below:

(1) $$\begin{array}{l} X=MJ+\epsilon \end{array}$$

where ***X*** ∈ ℝ^*C*×*T*^ is the EEG data comprising *C* channels and *T* time samples, ***J*** ∈ ℝ^*D*×*T*^ is the amplitude of *D* current dipoles distributed through the cortical surface with fixed orientation perpendicular to it, and ***M*** ∈ ℝ^*C*×*D*^ (termed *lead field matrix*) is the gain matrix that holds all available relationships between sources and EEG data influenced by a zero mean Gaussian noise **ϵ** ∈ ℝ^*C*×*T*^ with covariance $\text{cov}\left\{\epsilon \right\}=Q_{\epsilon }\in {\mathbb{R}}^{C\times C}$.

Since we assume ***J*** to be a zero mean Gaussian process that has prior covariance cov{***J***} = ***Q***_***J***_, with $Q_{J}\in {\mathbb{R}}^{D\times D}$, then, the brain activity estimation, $\hat{J},$ is carried out by solving the following optimization problem (Grech et al., 2008):

(2) $$\begin{array}{l} \hat{J}=\underset{J}{\text{argmin}}\left\{||X-MJ|{|}_{Q_{\epsilon }}^{2}+\lambda ||J|{|}_{Q_{J}}\right\}, \end{array}$$

where λ∈ℝ^+^ is the regularization parameter and notation $||\Pi |{|}_{\Delta }=\sqrt{\text{tr}\left\{\Pi^{⊤}\Delta^{-1}\Pi \right\}}$ stands for the Mahalanobis distance. Consequently, the first term relates to the quadratic error function while the second one introduces additional information for solving a given ill-posed task, usually, in a form of constraints that are imposed upon the source activity.

The inverse-problem optimization above-stated yields the following estimate:

$$\begin{array}{l} \hat{J}=Q_{J}M^{⊤}{\left(Q_{\epsilon }+MQ_{J}M^{⊤}\right)}^{-1}X \end{array}$$

This estimation demands some prior knowledge about the modeled sensor noise covariance ***Q***_**ϵ**_ and source covariance ***Q***_***J***_. To supply this information about the former matrix, we set ***Q***_**ϵ**_ = exp(λ_**ϵ**_)***I***_*C*_, where $I_{C}\in {\mathbb{R}}^{C\times C}$ is the appropriately sized identity matrix that is scaled by a hyper-parameter, modulating the sensor noise variance $\lambda_{\epsilon }\in {\mathbb{R}}^{+}$ (Phillips et al., 2002). In turn, the prior covariance matrix can be built as a sum over an assumed set that holds *P* spatial areas {***Q***_*p*_} so that each one relates to a single potentially activated cortex area, whose contribution to the mapped neural activity is expressed by the respective adding hyper-parameter, $\lambda_{p}\in {\mathbb{R}}^{+}$ (termed weight), as follows:

(3) $$\begin{array}{l} Q_{J}=\underset{p\in P}{\sum }\exp\left(\lambda_{p}\right)Q_{p}. \end{array}$$

The above prior covariance matrix gives rise to the possibility for implementing sparse-based approaches, in particular, the *Multiple Sparse Priors* (MSP).

In practice, the joint optimization of real-valued hyper-parameter sets {λ_**ϵ**_, λ_*p*_} can be accomplished by employing any standard variational scheme such as Expectation Maximization along with the greedy search algorithm (Belardinelli et al., 2012). Besides, the covariance components are also constructed so that each one regards a single locally smooth, focal patch of the cortex. That is, $Q_{p}=q_{p}{q_{p}}^{⊤}$, where $\left\{q_{p}\in {\mathbb{R}}^{D\times 1}\right\}$ is the set of non-overlapping patches that must cover the entire cortical surface. As suggested in Friston et al. (2008), the brain patch set are the columns of a Green's function matrix ***Q***_*G*_ = exp(σ***G***_*M*_), with $Q_{G}\in {\mathbb{R}}^{D\times D}$, where $G_{M}\in {\mathbb{R}}^{D\times D}$ is the graph Laplacian that comprises inter-dipole connectivity information about all neighboring dipoles. Here, the constant σ∈ℝ^+^ rules the smoothness of current distribution or spatial extent of the activated areas. As a rule, the graph Laplacian ***G***_*M*_ is calculated using an available head model through the adjacency matrix that incorporates all needed cortical information.

Moreover, a simpler approach, termed LORETA, can be directly formed by using the Green's function as the source prior covariance matrix, that it, by adjusting (Harrison et al., 2007)

(4) $$\begin{array}{l} Q_{J}=Q_{G} \end{array}$$

As a result, considering $j_{d}\in {\mathbb{R}}^{T\times 1}$ as the time course of the *d*−th dipole, where *d* = 1, …, *D*, the brain activity energy ē∈ℝ^*D*×1^ is reconstructed as follows:

$$\begin{array}{l} {ē}_{d}=𝔼\left\{j_{d}◦j_{d}:\forall t\in T\right\} \end{array}$$

where notations ◦ and 𝔼{·} stand for the Hadamard product and expectation operator, respectively.

### 2.2. Selection of spatially compact regions of interest

After computing the energy of brain activity, we focus on developing a data-driven approach to select the spatial ROI location accurately as to obtain the label vector $\iota \in \left[\iota_{d}\in {\mathbb{N}}^{N_{ROI}}\right]$ (with **ι** ∈ ℕ^*D*×1^) that better encodes the membership of each dipole to the set holding *N*_*ROI*_ ROIs, being ι_*d*_ the assigned label to the *d*–th dipole. For this purpose, we rely on the focal, powerful brain activity as an effective indicator of each region of interest, which we assume solely compact and without any space partition. Therefore, we take advantage of the already calculated brain activity reconstruction power $\bar{e}$ and the neighboring information available in the Green's function ***Q***_*G*_ to the select each ROI set element.

Concretely, all estimated brain activity energy is thoroughly scanned over the brain surface, i.e., {ē_*d*_ : ∀*d* ∈ *d*}, with the aim to detect the set of most powerful dipoles and their respective neighborhoods to avoid biasing the ROI estimates. This procedure is as follows: Firstly, we remove all power peaks that are below 10% of the amplitude activity with the maximum power. Consequently, those dipoles will not be labeled as members of any ROI. Then, we assume the first ROI as the neighboring spatial region that is enclosed within a prior fixed radius, ρ∈ℝ^+^, and centered at the dipole location that measures the most powerful neural activity. Likewise, the second ROI is further selected that we center at the dipole with next highest power, leaving out those dipoles that have been already labeled. This selection procedure is repeated until the whole set of most powerful dipoles is labeled, assuring that each one belongs to a single ROI. Note that the amount *N*_*ROI*_ is fixed automatically.

At this step, one label has been assigned to each dipole exceeding the prefixed threshold. Nevertheless, the ROI time courses estimation need to be addressed, as their quality determines the ROI-based feature selection and hence the extraction benefits. To this end, we average the estimated brain activity over the time courses, *j*_*d*_, that belong to the same ROI. So, the average over time courses is computed as:

$$\begin{array}{l} y_{r}=𝔼\left\{j_{d}\delta \left(\iota_{d}-r\right):\forall d=1,\ldots ,D\right\}, \end{array}$$

where δ() is the delta function. Thus, the matrix $Y\in {\mathbb{R}}^{N_{ROI}\times T}$ is built where each *r*−th row holds the corresponding ROI time series ***y***_*r*_, ∀*r*∈*N*_*ROI*_.

### 2.3. ROI-based feature extraction

Throughout this study, we consider three representative feature extraction methods that have been widely applied to emotion detection tasks (Alvarez-Meza et al., 2015):

#### 2.3.1. Power spectral density parameters

For the available ROI time series ***y***, we estimate the Power Spectral Density (PSD) noted as $s\in {\mathbb{R}}^{N_{B}},$ where *N*_*B*_∈ℕ is the number of frequency bins that is fixed according to the spectral band of interest, where the most discriminative information for MI is concentrated. Provided the EEG sample frequency $F_{s}\in {\mathbb{R}}^{+}$, the PSD vector ***s*** = {*s*_*f*_:*f* = 1, …, *N*_*B*_}, with *s*_*f*_∈ℝ and *N*_*B*_ = ⌊*F*_*s*_ ∕ 2⌋, is estimated by means of the nonparametric Welch's method that calculates the widely known Fast Fourier Transform algorithm of a set of *M*∈ℕ overlapping segments, which are split from the preprocessed EEG data vector. Due to the non-stationary nature of EEG data, the piecewise stationary analysis is carried out over the set of extracted overlapping segments that are further windowed by a smooth-time weighting window **α**∈ℝ^*L*^ that lasts *L*∈ℕ (*L*<*T*), yielding a set of the windowed segments {***v***^*m*^∈ℝ^*L*^:*m* = 1, …, *M*}, where $v_{i}^{m}\in \mathbb{R}$ (*i* = 1, …, *L*) is the *i*-th element of ***v***^*m*^. Consequently, the following modified periodogram vector $u=\left\{u_{f}\in {\mathbb{R}}^{+}\right\}$, $u\in {\mathbb{R}}^{N_{B}}$, is computed based on the Discrete Fourier Transform as follows:

$$\begin{array}{l} u_{f}=\underset{m\in M}{\sum }|\underset{i\in L}{\sum }v_{i}^{m}\exp\left(-j2\pi if\right){|}^{2}. \end{array}$$

Therefore, we extract each PSD element as *s*_*f*_ = *u*_*f*_ ∕ *M*ν, where the value $\nu =𝔼\left\{|\alpha_{i}{|}^{2}:\forall i\in L\right\}$.

#### 2.3.2. Wavelet-based parameters

Continuous Wavelet Transform (CWT) is a inner-product-based transformation quantifies similarity between a given equally sampled time series at time spacing δ_*t*_∈ℝ and a previously fixed base function γ(η), termed *mother wavelet* ruled by a dimensionless parameter vector η∈ℝ. Namely, each time element of the CWT vector **ς**^*g*^∈ℂ^*T*^ is extracted from the preprocessed EEG time-series ***z*** at scale *g*∈ℝ by accomplishing their convolution with the scaled and shifted mother wavelet in the form:

(5) $$\begin{array}{l} \varsigma_{t}^{g}=\underset{\tau \in T}{\sum }z_{\tau }\gamma^{*}\left(\left(\tau -t\right)\delta_{t}∕g\right), \end{array}$$

where (^*^) denotes the complex conjugate.

To effectively address the trade-off between time and frequency resolution in the non-stationary signal analysis, Discrete Wavelet Transform (DWT) had been developed that provides multi-resolution and non-redundant representation by decomposing the considered time-series into some sub-bands at different scales, yielding more precise time-frequency information about ***z***. Aiming to extract all suitable time-frequency information from DWT, the following detail vector ***b***^*j*^∈ℂ at level *j* is defined

(6) $$\begin{array}{l} b_{t}^{j}=\underset{k\in \mathbb{Z}}{\sum }a_{j,k}\psi_{j,k}\left(t\right), \end{array}$$

where *a*_*j, k*_ = ∑_*t*∈*T*_*z*_*t*_*h*_*j, k*_(*t*), with *a*_*j, k*_∈ℂ, *h*_*j, k*_(*t*)∈ℂ is the impulse response of a given wavelet filter. Then, provided a wavelet ψ(·), the DWT-based decomposition of ***z*** is computed as *z*_*t*_ = ∑_*j, k*∈ℤ_*a*_*j, k*_ ψ _*j, k*_(*t*).

#### 2.3.3. Hjorth parameters

For each windowed segment ***v***^*m*^, a set of the following three vector parameters describe EEG signals on the time domain:

(7a) $$\begin{array}{ll} Activity,\sigma_{v}^{2}\in {\mathbb{R}}^{M}: & \;\sigma_{m}^{2}=\mathrm{var}\left(v^{m}\right) \end{array}$$

(7b) $$\begin{array}{ll} Mobility,\phi_{v}\in {\mathbb{R}}^{M}: & \;\phi_{m}=\sqrt{\mathrm{var}\left(dv^{m}∕dt\right)∕\mathrm{var}\left(v^{m}\right)}, \end{array}$$

(7c) $$\begin{array}{ll} Complexity,\vartheta_{v}\in {\mathbb{R}}^{M}: & \;\vartheta_{m}=\phi_{m}^{\prime }∕\phi_{m} \end{array}$$

## 3. Experimental set-up

**Figure 1:**
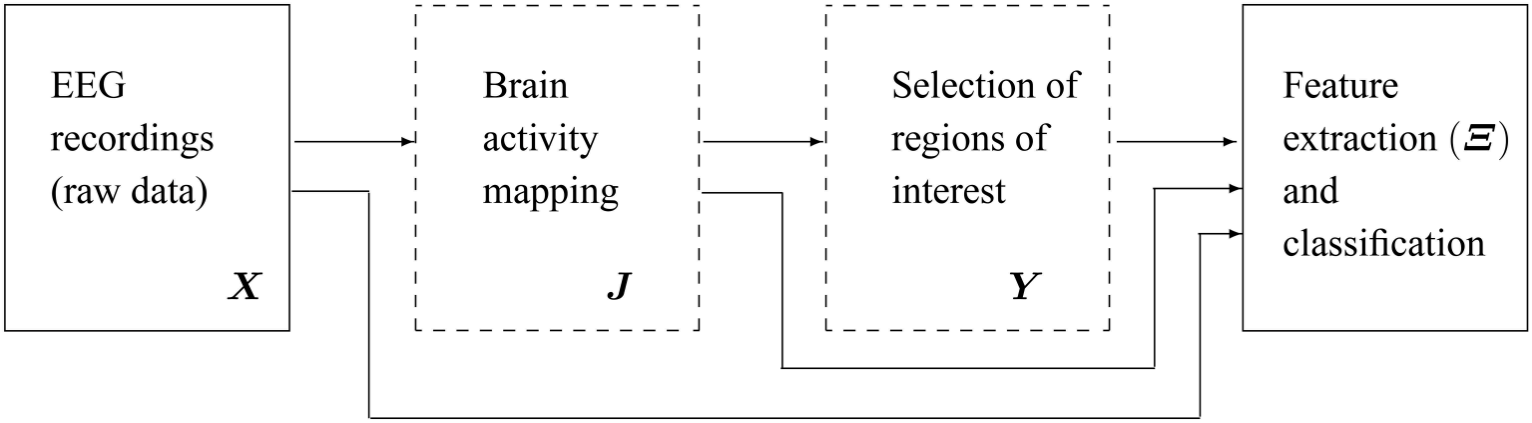
**Scheme of the proposed methodology for discrimination of emotions using extracted features from EEG source reconstruction**. The blocks marked in dashed lines are the subject of present study.

Figure 1 shows the main scheme of the proposed emotion discrimination using EEG source features. Owing to highlight the latent patterns in ***Y***, we propose a time-series discrimination methodology appraising the following stages: (i) EEG source estimation, (ii) Selection of Regions of interest (ROI), and (iii) feature extraction and classification.

### 3.1. Testing EEG database and preprocessing

The pre-processed EEG and subjective data used in the present study were obtained from the publicly available database for emotion analysis using physiological signals (DEAP) (Koelstra et al., 2012). Thirty-two healthy participants (50% females and 50% males in average aging 26.9 years) were recruited and consented to participate in the study. Thirty-two-channel EEG data were recorded using a Biosemi Active Two system. Data were acquired at a sampling rate 512 Hz, placing the electrodes on surface scalp according to the International 10-20 system. Pre-processing included the following steps: common referencing, down sampling to 128 Hz, high-pass filtering from 4 Hz, and eye blink artifact removal via independent component analysis.

All participants were presented with forty, one-minute long music videos with varying emotional content. Before every video, there was a baseline period of five seconds so that each participant was asked to fixate at across in the middle of screen. Following the presentation of each video, the participants were provided enough time to rate the music videos on a discrete 9-point scale for valence, arousal, dominance, and liking. Valence, arousal, and dominance dimensions were scored using the self-assessment manikins (SAM) to gouge user emotional states (Siegert et al., 2011). For liking (i.e., how much did you like the video?), thumbs up and thumbs down icons were used. Familiarity was rated after the end of the experiment on a 5-point integer scale (from “*never heard it before*” to “*listen to it regularly*”).

### 3.2. Brain activity mapping

As regards the inverse solution used for validation purpose, two approaches are performed: the baseline LORETA (see Equation 4) and MSP (see Equation 3) that are implemented using the SPM software. For the MSP approach, the employed spatial dictionary comprises 512 basis as to cover the whole cortical surface. Also, we fix the value for spatial coherence prior as σ = 0.6, which propagates spatial dependences over three or four mesh vertices that are, on average, about 6 mm apart to get a trade-off between spatial accuracy and local coherence.

**Figure 2:**
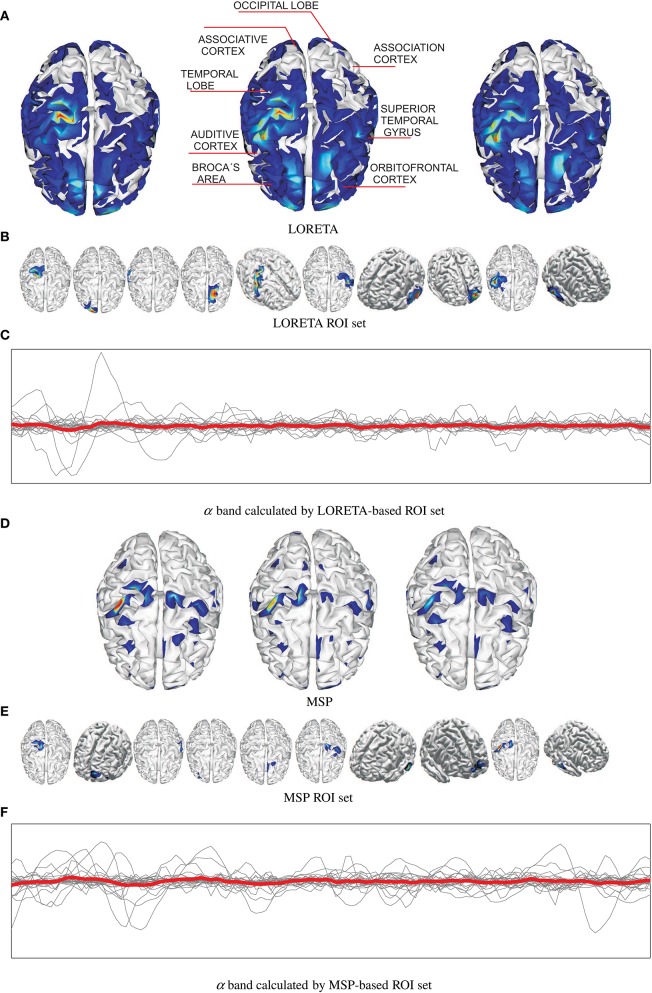
**Examples of estimated neural brain activity for N 27, trials labeled as #38, #39, and #40**. The activated regions are highlighted. **(A)** LORETA, **(B)** LORETA ROI set, **(C)** α band calculated by LORETA-based ROI set, **(D)** MSP, **(E)** MSP ROI set, **(F)** α band calculated by MSP-based ROI set.

Figure 2 shows an example of the reconstructed brain activity from EEG data using either tested approach of mapping for the subject N 27. For the sake of a better visual perception, the top row displays three examples of topographic figures that are obtained for the trials labeled as #38, #39, and #40. Aiming to get the physiological interpretation of estimated sources, the middle topographic also includes the most commonly referred brain activity areas related to emotions. Next, Figures 2A,D display the corresponding reconstructed neural activity using LORETA and MPS, respectively. The former mapping technique yields more blurred estimation producing wide zones (sometimes excessively) of cortical activation. In contrast, the MSP mapping results in more compact regions of estimated activation.

Upon the reconstructed neural activity, the ROI set is computed as shown in Figures 2B,E that displays the activated cortical locations over the topographic plots. So, both mappings discover some powerful common sources like somatosensory cortex, primary motor cortex, superior temporal gyrus, temporal lobe in the concrete case. However, the disparity between reconstructed neural activity brings two sets containing different activated areas. Thus, MSP finds considerable activity in the occipital lobe and orbitofrontal cortex, while LORETA detects in the association cortex. Therefore, the use of either mapping approach not necessarily leads to a unique ROI set.

Overall, the selected ROI set allows determining the location of those electrodes influencing the most, from which the features are to be extracted.

### 3.3. Feature extraction from selected ROI sets

For the sake of analysis, the feature set is extracted from three scenarios of input signal, namely: from the measured EEG channels, ***X***, the mapped activity ***J***, and the estimated ROI set ***Y***. So, the following parameters are fixed for each studied feature extraction method:

- *PSD-based and feature subsets:* For each band of interest (α, β, γ, and θ), the segment length value *L*, needed during calculation of the PSD and Hjort parameters, is adjusted as *L*>*F*_*r*_ ∕ *F*_*s*_, where *F*_*r*_ = 4 Hz is the smallest considered frequency. Accordingly, the calculated PSD features are the two first statistical moments of ***s*** computed for all bands.
- *WT-based feature subset:* A suitable wavelet function must be used to optimize the classifier performance. We select the Morlet wavelet for the CWT analysis because its wave shape and EEG signals are alike and it allows extracting features better localized in the frequency domain. Thus, we extract the short-time instantaneous CWT amplitudes using a set of the Morlet wavelets centered as follows: 2 Hz (to extract the δ band), 10 Hz (α band), 20 Hz (β band) and 50 Hz (γ band).

Figures 2C,F shows the trajectories of α band computed for either case of ROI set (subject N 27). Time series highlighted in red is the average as one of the features extracted. Table 1 shows the features extracted from EEG signals.

**Table 1:** **Amplitude estimators used as features extracted from EEG signals**.

**Parameter**	**Features**			**# Feat**
PSD	max(***s**_α_*)	𝔼{***s**_α_*}	var(***s**_α_*)	12
	max(***s**_β_*)	𝔼{***s**_β_*}	var(***s**_β_*)	
	max(***s**_γ_*)	𝔼{***s**_γ_*}	var(***s**_γ_*)	
	max(***s**_δ_*)	𝔼{***s**_δ_*}	var(***s**_δ_*)	
Hjort	max($\sigma_{v}^{2}$)	𝔼{$\sigma_{v}^{2}$}	var($\sigma_{v}^{2}$)	9
	max(**ϕ**_*v*_)	𝔼{**ϕ**_*v*_}	var(**ϕ**_*v*_)	
	max(**ϑ**_*v*_)	𝔼{**ϑ**_*v*_}	var(**ϑ**_*v*_)	
CWT	max(|**ς**^*g*_α_^|)	𝔼{|**ς**^*g*_α_^|}	var(|**ς**^*g*_α_^|)	12
	max(|**ς**^*g*_β_^|)	𝔼{|**ς**^*g*_β_^|}	var(|**ς**^*g*_β_^|)	
	max(|**ς**^*g*_γ_^|)	𝔼{|**ς**^*g*_γ_^|}	var(|**ς**^*g*_γ_^|)	
	max(|**ς**^*g*_δ_^|)	𝔼{|**ς**^*g*_δ_^|}	var(|**ς**^*g*_δ_^|)	
DWT	max(|***b***^*j*_α_^|)	𝔼{|***b***^*j*_α_^|}	var(|***b***^*j*_α_^|)	12
	max(|***b***^*j*_β_^|)	𝔼{|***b***^*j*_β_^|}	var(|***b***^*j*_β_^|)	
	max(|***b***^*j*_γ_^|)	𝔼{|***b***^*j*_γ_^|}	var(|***b***^*j*_γ_^|)	
	max(|***b***^*j*_δ_^|)	𝔼{|***b***^*j*_δ_^|}	var(|***b***^*j*_δ_^|)	
Total features				45

# Feat, number of features.

### 3.4. Classifier training and validation

In this stage, the statistical measures of the short-time parameters are computed, as described in Section 2, to extract the input feature space matrix **Ξ** = {**ξ**_*n*_:*n* = 1, …, *N*_*tr*_} with $\Xi \in {\mathbb{R}}^{N_{tr}\times N_{F}}$. Hence, the row vector $\xi_{n}\in {\mathbb{R}}^{N_{F}}$ holds *N*_*F*_ = *C*×*F* concatenated features for the *n*-th trial, being *N*_*F*_∈ℕ the number of provided features of the *tr*-th trial for a given channel. Here, *C* = 32, *F* = 45, and *N*_*tr*_ = 40.

Before accomplishing the classification stage, a stochastic relevance analysis of the extracted feature set **Ξ** is performed by means of its eigenvalues and eigenvectors. Consequently, the input features are ranked following the yielded relevance vector, as described in detail in Alvarez-Meza et al. (2015). The main assumption behind this relevance analysis is that the largest values of the ranking vector should point out to the better input attributes since they exhibit higher overall correlations to the estimated principal components. The variance explained is adjusted to 90% for mapping the most relevant components.

Additionally, the set of relevant features feeds a soft-margin support vector machine (SVM) based classifier that is trained in the following leave-one-out cross-validation methodology: (i) D-1 samples in the database are used to learn the most relevant features and to train the classifier, while the remaining sample is used for testing, (ii) repeat the preceding stage until all samples have been used as testing sample. This procedure is implemented to solve the four different binary classification problems: low/high arousal, low/high dominance, low/high valence and low/high liking. Note that the reason for choosing a leave-one-sample-out cross-validation scheme is that the number of samples per subject is not enough for generating significant training and testing sets, making this strategy the most used in emotion discrimination for the DEAP database.

With the aim to configure the low and high classes, the subjective scores are thresholded at the mid-point of a 9-pointscale, i.e., at 5. Since this procedure results in unbalanced classes for each subjective rating, the F1-score, along with classification accuracies, is employed to describe reliably the results of classifier performance while tackling the class imbalance, as suggested in Gupta et al. (2016).

**Figure 3:**
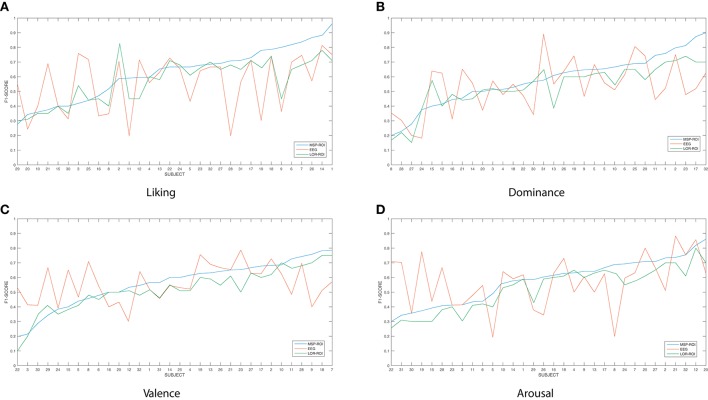
**Performed accuracy by the considered experiment setups for all considered emotions**. The cardinal of subjects is rearranged following the obtained accuracy by MSP-ROI, though the original database cardinal is shown. **(A)** Liking, **(B)** Dominance, **(C)** Valence, **(D)** Arousal.

Figure 3 shows the accuracy performed by the SVM classifier for each one of the considered emotions. In terms of feature extraction, there are three experiment setups of emotion discrimination: Features extracted directly from the EEG data, avoiding any mapping activity (noted as EEG), features from ROI sets computed for LORETA (LOR-ROI) and MSP (MSP-ROI) reconstruction techniques. For a better illustration, the cardinal of subjects is rearranged following the obtained accuracy by the proposed extraction of selected ROI time series (i.e., the MSP-ROI experiment). All subjects are ranked by accuracy score in decreasing order.

Figure 3A shows the performed F1 values for liking discrimination that has been widely reported as the emotion having the most complex random structure. The use of features extracted from spatially compact ROI set results in a higher accuracy achieved by either tested approach of brain activity mapping. For most of the subjects, the MSP-ROI approach overcomes LOR-ROI and EEG settings. Although the same situation holds for the Dominance state, the advantage of MSP-ROI performance somewhat reduces (see Figure 3B). Further, in the case of Valence discrimination, MSP-ROI also performs the best, but the LOR-ROI behaves very close to the plain EEG (see Figure 3C). Lastly, neither ROI-based strategy seems to outperform the plain EEG for Arousal emotion (see Figure 3D).

To compare all considered approaches of neural analysis, we quantify the statistical difference regarding the F1 score and classifier accuracy *a*_*c*_, validating whether the MSP-ROI performance over subjects is higher than the one obtained by other approaches. To this end, a paired sample *t*-test is carried out in which the null hypothesis states that there are not significant differences between MSP-ROI and each compared approach in terms of the performed population mean values. Otherwise, the alternative hypothesis states that our population mean are greater that the other our population-means calculated over all subjects are greater that other values.

Table 2 summarizes the achieved performance by each examined training setup, regarding the maximum and average values for all subjects.Superscripted stars indicate whether the population mean of MSP-ROI is greater than the population mean of EEG (taking into account accuracy and F1-score separately ^**^ = *p* < 0.01, ^*^ = *p* < 0.05). Subscripted diamonds indicate whether the population mean of MSP-ROI is greater than the population mean of LOR-ROI (taking into account accuracy and F1-score separately ◇◇ = *p* < 0.01, ◇ = *p* < 0.05). From the obtained results for either performance measure (F1 score and accuracy), it can be noted that the use of MSP mapping allows increasing the system accuracy so that it even outperforms LORETA in either case of setting regardless the discriminating emotion. Therefore, the incorporation of features, extracted from spatially compact Regions of Interest, enables to enhance further the performed accuracy of emotion discrimination.

**Table 2:** **Classification performance (F1 Score and Accuracy)**.

	**Liking**		**Dominance**		**Valence**		**Arousal**	
	**Max**	**Mean**	**Max**	**Mean**	**Max**	**Mean**	**Max**	**Mean**
EEG(F1)	81.93	56.05 ± 9.6	88.5	52.9 ± 9.1	78.69	54.86 ± 9.3	88.23	57.64 ± 8.8
EEG(Acc)	72.5	51.85 ± 12.6	80.1	51.4 ± 12.1	77.59	55.76 ± 9.3	80.23	53.74 ± 8.8
LOR-ROI (F1)	78.5	56.8 ± 8.1	76.3	53.2 ± 9.1	74.3	54.9 ± 10.6	81.2	53.8 ± 10.2
LOR-ROI(Acc)	73.1	54.5 ± 10.1	71.2	51.9 ± 11.2	72.9	53.2 ± 8.4	73.41	56.6 ± 10.1
MSP-ROI (F1)	96.2	(62.5 ± 9.7)_◇_^**^	86.4	(59.4 ± 9.6)_◇_^**^	75.4	(60.8 ± 10.1)_◇__◇_^**^	85.6	(60.3 ± 8.5)_◇_^**^
MSP-ROI (Acc)	92.7	(57.3 ± 13.9)_◇__◇_^**^	75.3	(58.3 ± 9.3)_◇__◇_	72.1	(58.6 ± 10.6)^*^	77.6	(52.8 ± 11.6)_◇_^*^

## 4. Discussion and concluding remarks

We propose a method for supporting discrimination of emotions that employs features extracted from spatially compact regions of interest. To this end, we aim to compute the characterizing ROI set upon the reconstructed brain neural activity, using EEG recordings.

**Figure 4:**
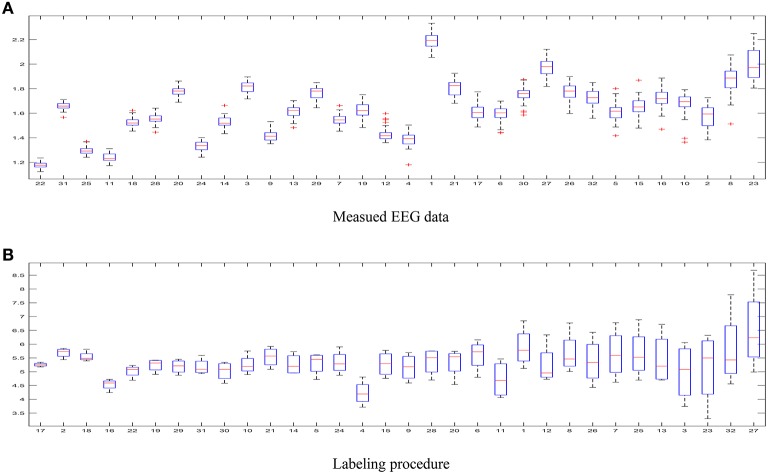
**Uncertainty estimated for the DEAP database**. **(A)** Measued EEG data, **(B)** Labeling procedure.

However, during training and validation of the proposed discrimination method, the following factors are to be considered:

– The first aspect to reflect is the influence of intrinsic uncertainty provided by the acquired EEG data. As seen in Figure 4A that shows the entropy as measure of uncertainty, the utmost disparity between subjects is as much as ten times. In fact, some of the outlier recordings (v.g. #27, #8, and #23) are quite noisy. However, there is another source of great incertitude that is related to the labeling procedure used during construction of databases for affect representation and recognition. Thus, Figure 4B displays the ranked values of entropy for the DEAP label set. As already discussed in Verma and Tiwary (2016), the large value of standard deviation for each emotion points out on their labeling difficulty.
– The following aspect concerns the technique that we apply for imaging EEG activity. The first tested method of neural mapping is LORETA that has the characteristic that localization is preserved with a certain amount of dispersion, i.e., it has a relatively low spatial resolution (Pascual-Marqui et al., 1994). Consequently, the reconstructed brain activity is more blurred, producing wide zones (sometimes excessively) of cortical activation. In contrast, the use of sparse-based approaches (like MSP) notably improves identification of the source signals from noisy electroencephalographic measurements (Becker et al., 2015). As a result, MSP allows performing brain source imaging so that we obtain more compact regions of activation. Furthermore, due to the low spatial resolution, Loreta algorithm frequently infers false regions of activation, where powerful common sources should not be present. Therefore, although LORETA has been widely used in the last years to localize electrical generators of scalp EEG data, its confidence of estimated areas of activation may be not enough.
– Generally speaking, a challenging issue relating to the emotion detection is how to identify ROI sets precisely at very short temporal scales; this dilemma remains common for all cognitive tasks (Hassan et al., 2015). Similar studies have selected the ROIS set by identifying particular gyral landmarks on subject specific cortex models (Mattia et al., 2006). However, we rather employ a data-driven approach that focuses on accurately encoding the membership of each dipole to the estimated areas with salient cortical activation (identified as the Region-of-Interest set) so that every single ROI is assumed solely compact and without any space partition. Thus, each selected ROI set allows considering the time series of neural activity, contributing the most to the emotion states. As a result, the introduced ROI sets enhance the performed detection accuracy.
– We carry out the binary classification as the only one reported in the literature for emotion recognition. However, the use of hard thresholding algorithms for binarizing a label set leads to losing most of the emotional richness. Moreover, the scores near the midpoints and extreme values may have different implications. Therefore, other strategies of labeling should be considered to better capture the richness of emotion dimensions like the use of regression.
– The next factor of value is the classifier setup. We use SVM classifier with radial basis kernel that is assumed more robust to class-imbalance of training emotion data (Daimi and Saha, 2014). For this purpose, we consider three representative feature extraction methods that have been widely applied to emotion detection tasks. However, due to the high dimension of the input feature space, feature selection is carried out trough PCA before feeding the classifier. As a result, we obtain a reduced set of features that represent the most the properties of input training space.
– Lastly, Table 3 summarizes the comparison of emotion identification systems that had been recently reported in the literature, regarding two commonly used performance measures: F1 and accuracy. As seen, although the proposed approach obtains competitive scores of accuracy for the same dataset used here, other methods are performing higher values employing similar features and classifiers. Nonetheless, the discussed MSP-ROI method reaches the biggest F1 values for all considered emotion classes. This matter deserves particular attention because of the imbalanced class of validated database, making the F1-score more reliable to quantify the classifier performance in this study (Gupta et al., 2016). Note that the obtained results agree with those studies in BCI areas like in Edelman et al. (2015), where the use of spatially compact regions of interest have also been applied, allowing to better identify the network activity of these tasks in source space.

**Table 3:** **Performance comparison reported in the literature based on EEG features**.

**Features**	**Classifier**	**Valence**		**Arousal**		**Dominance**		**Liking**		**References**
		**F1**	***a*_*c*_**	**F1**	***a*_*c*_**	**F1**	***a*_*c*_**	**F1**	***a*_*c*_**	
PSD, AI	NB	56	57	58	62	–	–	50	55	Koelstra et al., 2012+
PSD	SVM	50	50	60	62	–	–	–	–	Soleymani et al., 2012
PSD, AI	SVM	49	61	56	57	52	53	54	64	Daimi and Saha, 2014+
DT-CWT	SVM	55	**65**	57	**67**	55	**69**	50	**71**	Daimi and Saha, 2014+
PSD	SVM	59	60	60	60	58	58	59	60	Gupta et al., 2016+
PSD,HJORT										
CWT,DWT	SVM	**60.8**	58.6	**60.3**	52.8	**59.4**	58.3	**62.5**	57.3	Here

Notation stands for non reported results. Works marked with ^+^ employ the same dataset used here. PSD, Power spectral density; AI, Asymmetry Index; DT−CWT, Dual Tree Complex Wavelet Packet Transform; SVM, Support Vectors Machines; NB, Naive Baye's. Bold means better results.

Therefore, we conclude that incorporation of the proposed training method in combination with enhanced spatial resolution allows improving the performed accuracy of discrimination in most of the considered emotions, namely: dominance, valence, and liking.

As future work, the authors intend to refine the recognition method by investigating a finer and more complex class of emotions. Besides, the use of more elaborate methods for extraction of temporal information from the ROI set is to be strongly considered such as the connectivity analysis. Another, aspect of improving is the feature selection that should take into consideration the nonstationary behavior of EEG data.

## Author contributions

JP: His research interests are feature extraction/selection for training pattern recognition systems, bio-engineering, neuroscience and machine learning. JM: His research interests include machine learning and signal processing methods applied to image and video data analysis as well as bioengineering tasks. GC: His teaching and research interests include information and signal theory, digital signal processing and bioengineering.

### Conflict of interest statement

The authors declare that the research was conducted in the absence of any commercial or financial relationships that could be construed as a potential conflict of interest. The handling Editor declared a shared affiliation, though no other collaboration, with one of the authors JIP-B and states that the process nevertheless met the standards of a fair and objective review.

## References

[B1] Agrafioti F. Hatzinakos D. Anderson A. (2012). Ecg pattern analysis for emotion detection. IEEE Trans. Affect. Comput. 3, 102–115. 10.1109/T-AFFC.2011.28

[B2] Ahammed K. (2015). Identification of human emotions via univariate and multivarite multiscale entropy. Am. J. Eng. Appl. Sci. 8, 410–416. 10.3844/ajeassp.2015.410.416

[B3] Alvarez-Meza A. Velasquez-Martinez L. Castellanos-Dominguez G. (2015). Time-series discrimination using feature relevance analysis in motor imagery classification. Neurocomputing 151(Pt 1):122–129. 10.1016/j.neucom.2014.07.077

[B4] Becker H. Albera L. Comon P. Gribonval R. Wendling F. Merlet I. (2015). Brain-source imaging: from sparse to tensor models. IEEE Signal Process Mag. 32, 100–112. 10.1109/MSP.2015.2413711

[B5] Belardinelli P. Ortiz E. Barnes G. Noppeney U. Preissl H. (2012). Source reconstruction accuracy of MEG and EEG bayesian inversion approaches. PLoS ONE 7:e51985. 10.1371/journal.pone.0051985 23284840 PMC3527408

[B6] Brookes M. J. O'Neill G. C. Hall E. L. Woolrich M. W. Baker A. Corner S. P.. (2014). Measuring temporal, spectral and spatial changes in electrophysiological brain network connectivity. NeuroImage 91, 282–299. 10.1016/j.neuroimage.2013.12.066 24418505

[B7] Castaño-Candamil S. Höhne J. Martínez-Vargas J.-D. An X.-W. Castellanos-Domínguez G. Haufe S. (2015). Solving the EEG inverse problem based on space-time-frequency structured sparsity constraints. NeuroImage 118, 598–612. 10.1016/j.neuroimage.2015.05.052 26048621

[B8] Daimi S. N. Saha G. (2014). Classification of emotions induced by music videos and correlation with participants' rating. Exp. Syst. Appl. 41, 6057–6065. 10.1016/j.eswa.2014.03.050

[B9] Dan-Glauser E. S. Gross J. J. (2015). The temporal dynamics of emotional acceptance: experience, expression, and physiology. Biol. Psychol. 108, 1–12. 10.1016/j.biopsycho.2015.03.005 25782407

[B10] Edelman B. Baxter B. He B. (2015). Decoding and mapping of right hand motor imagery tasks using EEG source imaging, in 2015 7th International IEEE/EMBS Conference on Neural Engineering (NER) (Montpellier: IEEE), 194–197. 10.1109/NER.2015.7146593

[B11] Edelman B. Baxter B. He B. (2016). EEG source imaging enhances the decoding of complex right-hand motor imagery tasks. IEEE Trans. Biomed. Eng. 63, 4–14. 10.1109/TBME.2015.2467312 26276986 PMC4716869

[B12] Friston K. Harrison L. Daunizeau J. Kiebel S. Phillips C. Trujillo-Barreto N.. (2008). Multiple sparse priors for the M/EEG inverse problem. NeuroImage 39, 1104–1120. 10.1016/j.neuroimage.2007.09.048 17997111

[B13] Grech R. Cassar T. Muscat J. Camilleri K. Fabri S. Zervakis M.. (2008). Review on solving the inverse problem in EEG source analysis. J. NeuroEng. Rehabil. 5, 792–800. 10.1186/1743-0003-5-25 18990257 PMC2605581

[B14] Gupta R. Laghari K. R. Falk T. H. (2016). Relevance vector classifier decision fusion and EEG graph-theoretic features for automatic affective state characterization. Neurocomputing 174(Pt B):875–884. 10.1016/j.neucom.2015.09.085

[B15] Harrison L. Penny W. Ashburner J. Trujillo-Barreto N. Friston K. (2007). Diffusion-based spatial priors for imaging. NeuroImage 38, 677–695. 10.1016/j.neuroimage.2007.07.032 17869542 PMC2643839

[B16] Hassan M. Dufor O. Benquet P. Berrou C. Wendling F. (2015). Identification of brain networks with high time/space resolution using dense EEG, in 2015 7th International IEEE/EMBS Conference on Neural Engineering (NER) (Montpellier: IEEE), 1060–1063. 10.1109/NER.2015.7146810

[B17] Jirayucharoensak S. Pan-Ngum S. Israsena P. (2014). EEG-based emotion recognition using deep learning network with principal component based covariate shift adaptation. Sci. World J. 2014:627892. 10.1155/2014/627892 25258728 PMC4165739

[B18] Kober H. Barrett L. F. Joseph J. Bliss-Moreau E. Lindquist K. Wager T. D. (2008). Functional grouping and cortical-subcortical interactions in emotion: a meta-analysis of neuroimaging studies. NeuroImage 42, 998–1031. 10.1016/j.neuroimage.2008.03.059 18579414 PMC2752702

[B19] Koelstra S. Muhl C. Soleymani M. Lee J.-S. Yazdani A. Ebrahimi T. (2012). Deap: a database for emotion analysis; using physiological signals. IEEE Trans. Affec. Comput. 3, 18–31. 10.1109/T-AFFC.2011.15

[B20] Illán I. A. Górriz J. M. López M. M. Ramírez J. Salas-Gonzalez D. Segovia F. (2011). Computer aided diagnosis of Alzheimer's disease using component based SVM. Appl. Soft Comput. 11, 2376–2382. 10.1016/j.asoc.2010.08.019

[B21] Liu Y. Sourina O. (2013). EEG databases for emotion recognition, in 2013 International Conference on Cyberworlds (CW) (Yokohama), 302–309. 10.1109/CW.2013.52

[B22] Liu Y.-H. Cheng W.-T. Hsiao Y.-T. Wu C.-T. Jeng M.-D. (2014). Eeg-based emotion recognition based on kernel fisher's discriminant analysis and spectral powers, in 2014 IEEE International Conference on Systems, Man and Cybernetics (SMC) (San Diego, CA), 2221–2225. 10.1109/SMC.2014.6974254

[B23] López M. Ramírez J. Górriz J. M. Alvarez I. Salas-Gonzalez D. Segovia F. (2011). Principal component analysis-based techniques and supervised classification schemes for the early detection of alzheimer's disease. Neurocomputing 74, 1260–1271. 10.1016/j.neucom.2010.06.025

[B24] Marrero-Fernandez P. Montoya-Padron A. i Capo A. J. Rubio J. M. B. (2014). Evaluating the research in automatic emotion recognition. IETE Tech. Rev. 31, 220–232. 10.1080/02564602.2014.906863

[B25] Mattia D. Cincotti F. Mattiocco M. Scivoletto G. Marciani M. G. Babiloni F. (2006). Motor-related cortical dynamics to intact movements in tetraplegics as revealed by high-resolution EEG. Hum. Brain Mapp. 27, 510–519. 10.1002/hbm.20195 16124014 PMC6871478

[B26] Muthu Meena S. Vimala K. Kalaivani V. (2015). Emotional stress recognition using multi-modal bio-signals. Biomet. Bioinform. 7, 17–22.

[B27] Nordhjem B. Curcic-Blake B. J. T. Meppelink A. M. Renken R. J. De Jong B. M. Leenders K. L.. (2016). Lateral and medial ventral occipitotemporal regions interact during the recognition of images revealed from noise. Front. Hum. Neurosci. 9:678. 10.3389/fnhum.2015.00678 26778997 PMC4701927

[B28] Pascual-Marqui R. Michel C. Lehmann D. (1994). Low resolution electromagnetic tomography: a new method for localizing electrical activity in the brain. Int. J. Psychophysiol. 18, 49–65. 10.1016/0167-8760(84)90014-X 7876038

[B29] Phillips C. Rugg M. D. Friston K. J. (2002). Systematic regularization of linear inverse solutions of the EEG source localization problem. NeuroImage 17, 287–301. 10.1006/nimg.2002.1175 12482084

[B30] Robinson I. B. Robinson M. D. (2009). Measures of emotion: a review. Cogn. Emot. 23, 209–237. 10.1080/02699930802204677 19809584 PMC2756702

[B31] Schoffelen J.-M. Gross J. (2009). Source connectivity analysis with MEG and EEG. Hum. Brain Mapp. 30, 1857–1865. 10.1002/hbm.20745 19235884 PMC6870611

[B32] Siegert I. Bock R. Vlasenko B. Philippou-Hubner D. Wendemuth A. (2011). Appropriate emotional labelling of non-acted speech using basic emotions, geneva emotion wheel and self assessment manikins, in 2011 IEEE International Conference on Multimedia and Expo (ICME) (Barcelona), 1–6. 10.1109/ICME.2011.6011929

[B33] Sirca F. Onorati F. Mainardi L. Russo V. (2015). Time-varying spectral analysis of single-channel EEG: application in affective protocol. J. Med. Biol. Eng. 35, 367–374. 10.1007/s40846-015-0044-5

[B34] Soleymani M. Pantic M. Pun T. (2012). Multimodal emotion recognition in response to videos. IEEE Trans. Affect. Comput. 3, 211–223. 10.1109/T-AFFC.2011.37

[B35] Toppi J. Astolfi L. Poudel G. R. Innes C. R. Babiloni F. Jones R. D. (2016). Time-varying effective connectivity of the cortical neuroelectric activity associated with behavioural microsleeps. NeuroImage 124, 421–432. 10.1016/j.neuroimage.2015.08.059 26363348

[B36] Verleysen M. François D. (2005). The curse of dimensionality in data mining and time series prediction, in Computational Intelligence and Bioinspired Systems, eds Cabestany J. Prieto A. Sandoval F. (Berlin; Heidelberg: Springer), 758–770. 10.1007/11494669_93

[B37] Verma G. K. Tiwary U. S. (2016). Affect representation and recognition in 3d continuous valence–arousal–dominance space. Multi. Tools Appl. 1–25. 10.1007/s11042-015-3119-y

[B38] Virvou M. Tsihrintzis G. A. Alepis E. Stathopoulou I.-O. Kabassi K. (2015). Intelligent Interactive Multimedia Systems and Services in Practice, Chapter On the Use of Multi-attribute Decision Making for Combining Audio-Lingual and Visual-Facial Modalities in Emotion Recognition Cham: Springer International Publishing.

[B39] Wang X.-W. Nie D. Lu B.-L. (2014). Emotional state classification from EEG data using machine learning approach. Neurocomputing 129, 94–106. 10.1016/j.neucom.2013.06.046

[B40] Wentzel K. R. Wigfield A. (2009). Handbook of Motivation at School. New York, NY: Routledge.

